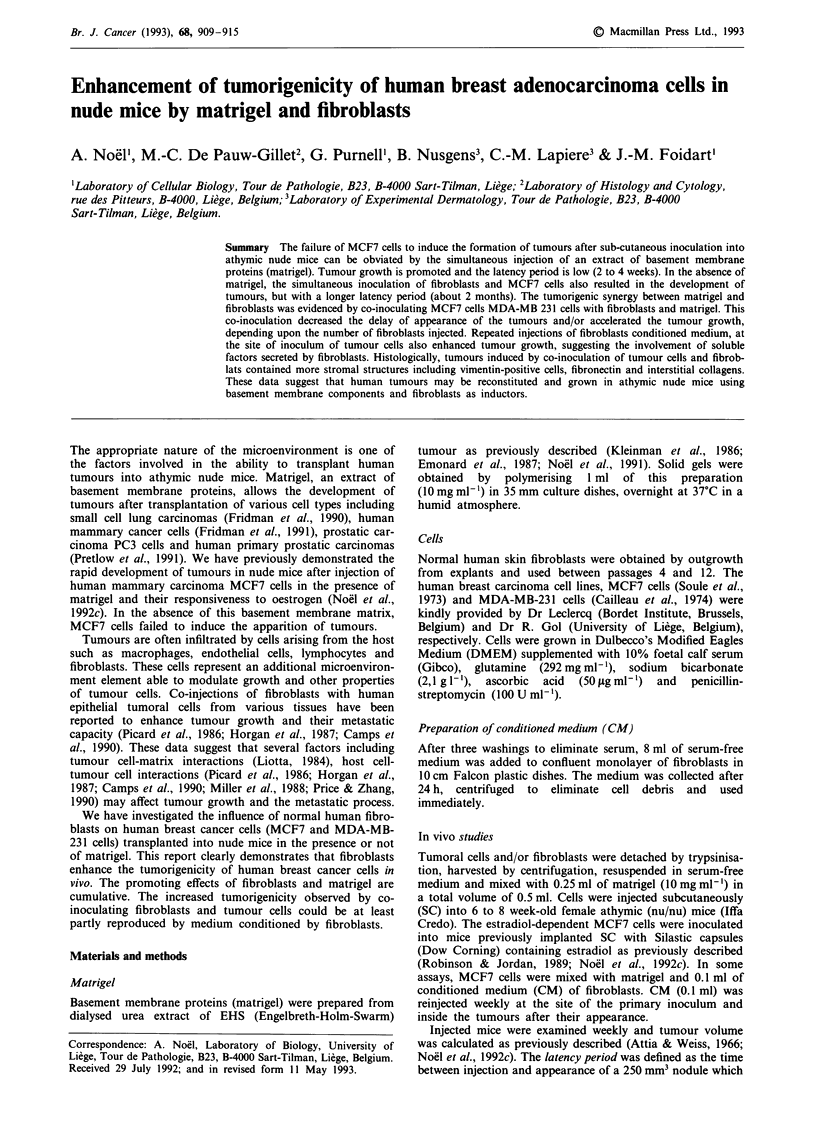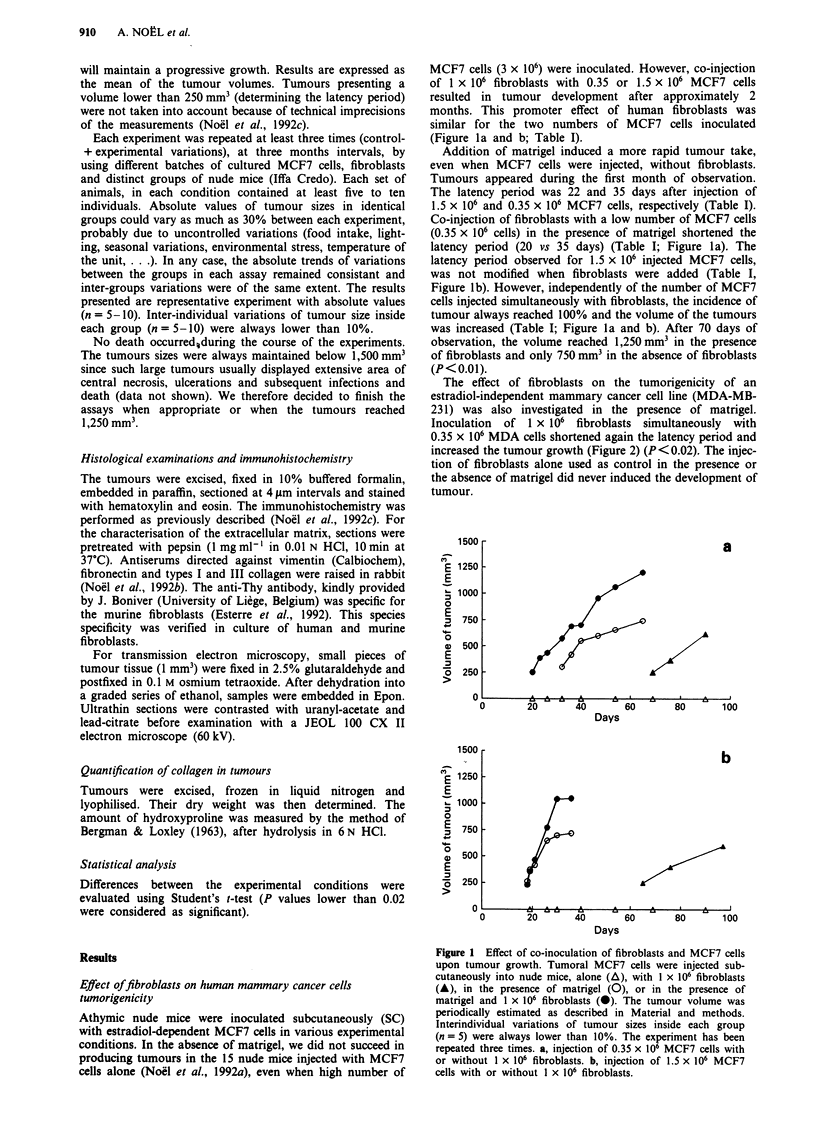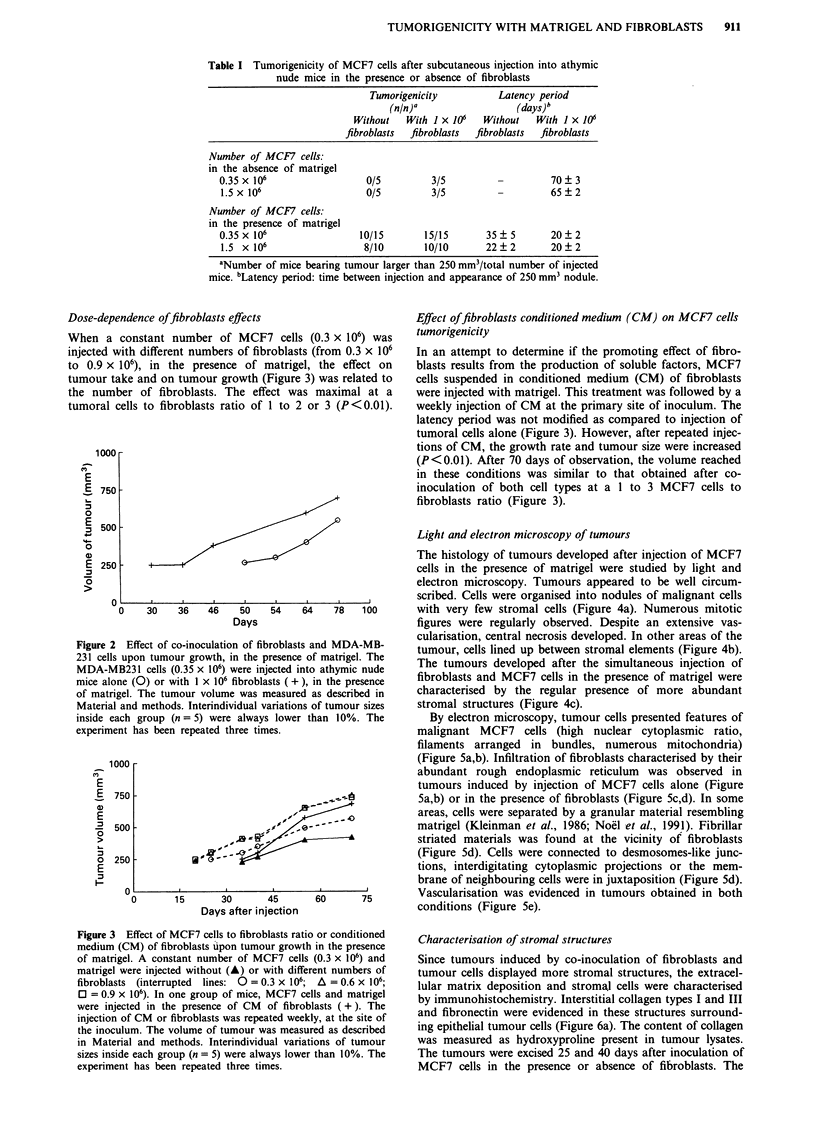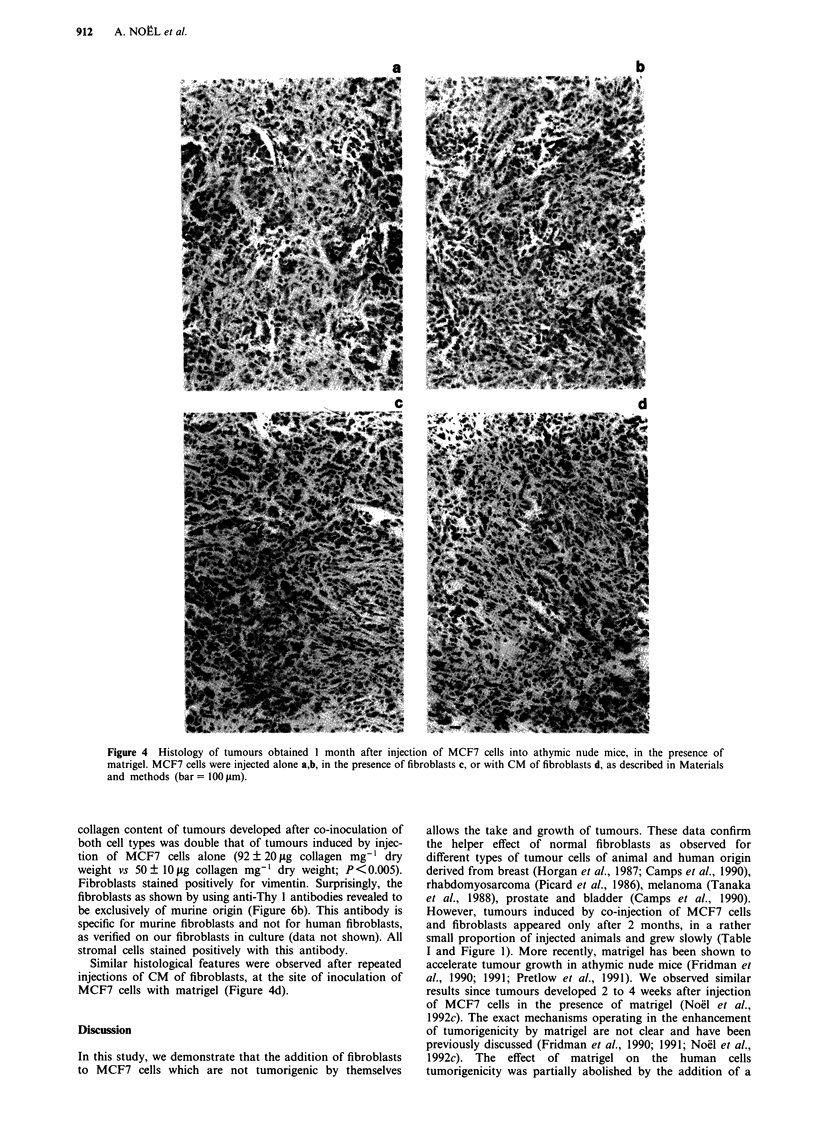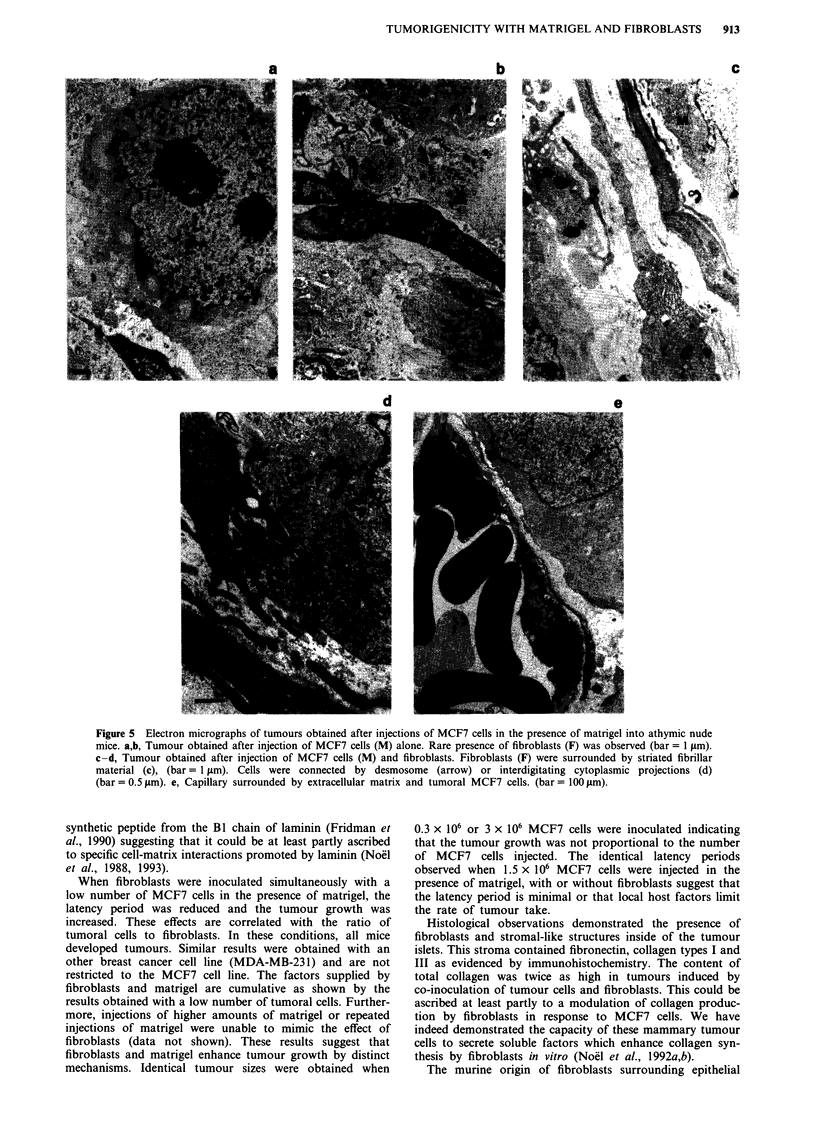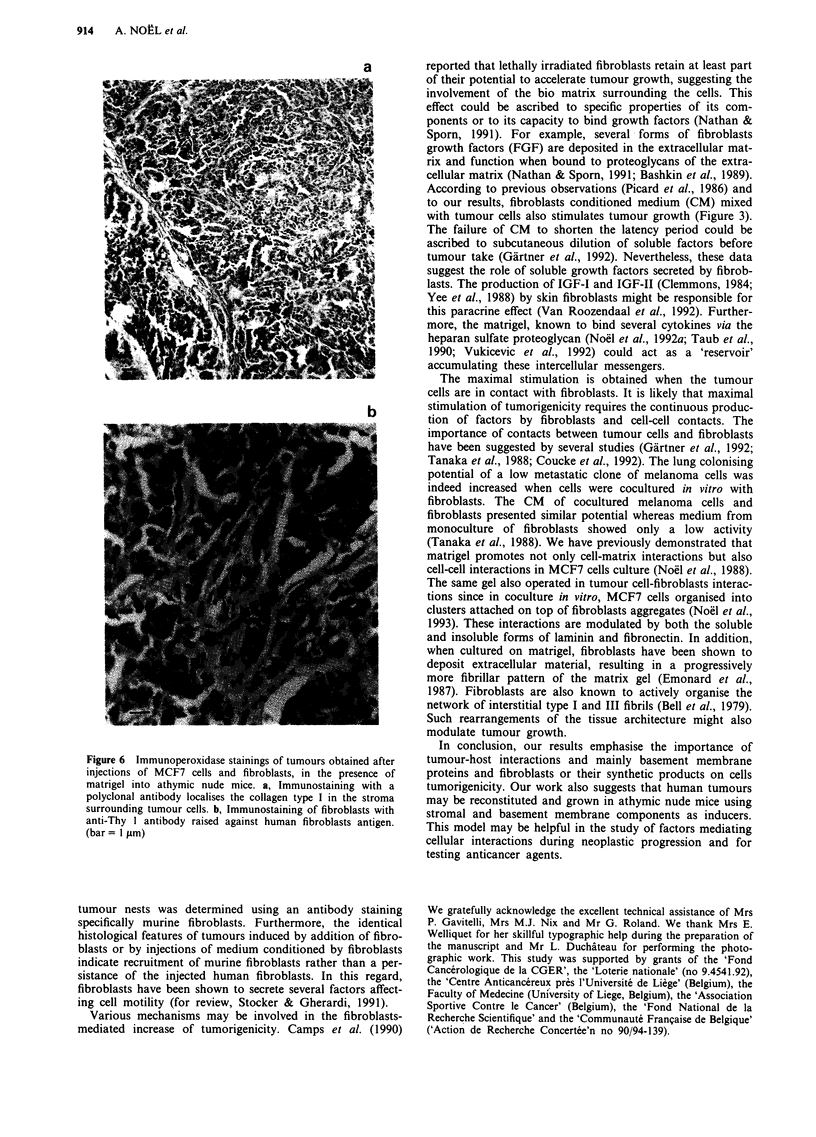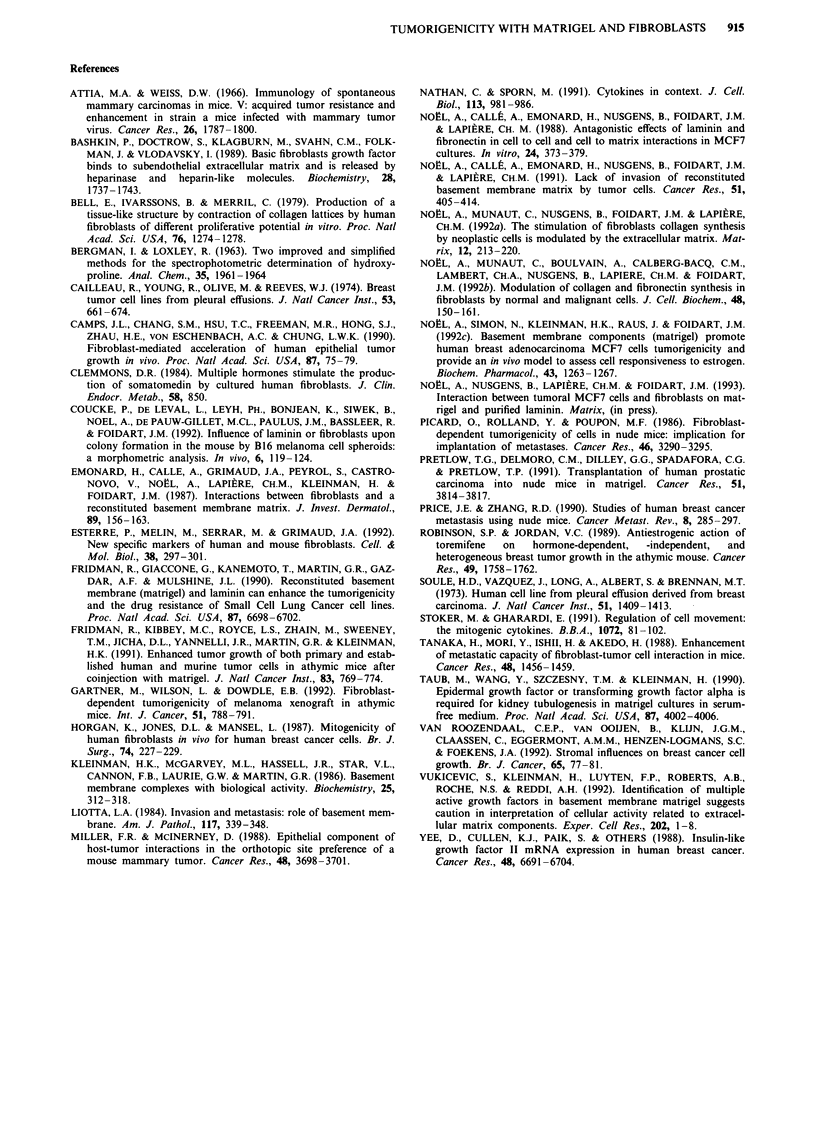# Enhancement of tumorigenicity of human breast adenocarcinoma cells in nude mice by matrigel and fibroblasts.

**DOI:** 10.1038/bjc.1993.453

**Published:** 1993-11

**Authors:** A. Noël, M. C. De Pauw-Gillet, G. Purnell, B. Nusgens, C. M. Lapiere, J. M. Foidart

**Affiliations:** Laboratory of Cellular Biology, Tour de Pathologie, Sart-Tilman, Liège, Belgium.

## Abstract

**Images:**


Br. J. Cancer (1993), 68, 909-915 © Macmillan Press Ltd., 1993

### Enhancement of tumorigenicity of human breast adenocarcinoma cells in nude mice by matrigel and fibroblasts

A. Noël[1], M.-C. De Pauw-Gillet[2], G. Purnell[1], B. Nusgens[3], C.-M. Lapiere[3] & J.-M. Foidart[1]

[1]*Laboratory of Cellular Biology, Tour de Pathologie, B23, B-4000 Sart-Tilman, Liège;* [2]*Laboratory of Histology and Cytology, rue des Pitteurs, B-4000, Liège, Belgium;* [3]*Laboratory of Experimental Dermatology, Tour de Pathologie, B23, B-4000 Sart-Tilman, Liège, Belgium.*

**Summary** The failure of MCF7 cells to induce the formation of tumours after sub-cutaneous inoculation into athymic nude mice can be obviated by the simultaneous injection of an extract of basement membrane proteins (matrigel). Tumour growth is promoted and the latency period is low (2 to 4 weeks). In the absence of matrigel, the simultaneous inoculation of fibroblasts and MCF7 cells also resulted in the development of tumours, but with a longer latency period (about 2 months). The tumorigenic synergy between matrigel and fibroblasts was evidenced by co-inoculating MCF7 cells MDA-MB 231 cells with fibroblasts and matrigel. This co-inoculation decreased the delay of appearance of the tumours and/or accelerated the tumour growth, depending upon the number of fibroblasts injected. Repeated injections of fibroblasts conditioned medium, at the site of inoculum of tumour cells also enhanced tumour growth, suggesting the involvement of soluble factors secreted by fibroblasts. Histologically, tumours induced by co-inoculation of tumour cells and fibroblats contained more stromal structures including vimentin-positive cells, fibronectin and interstitial collagens. These data suggest that human tumours may be reconstituted and grown in athymic nude mice using basement membrane components and fibroblasts as inductors.

The appropriate nature of the microenvironment is one of the factors involved in the ability to transplant human tumours into athymic nude mice. Matrigel, an extract of basement membrane proteins, allows the development of tumours after transplantation of various cell types including small cell lung carcinomas (Fridman *et al.*, 1990), human mammary cancer cells (Fridman *et al.*, 1991), prostatic carcinoma PC3 cells and human primary prostatic carcinomas (Pretlow *et al.*, 1991). We have previously demonstrated the rapid development of tumours in nude mice after injection of human mammary carcinoma MCF7 cells in the presence of matrigel and their responsiveness to oestrogen (Noël *et al.*, 1992*c*). In the absence of this basement membrane matrix, MCF7 cells failed to induce the apparition of tumours.

Tumours are often infiltrated by cells arising from the host such as macrophages, endothelial cells, lymphocytes and fibroblasts. These cells represent an additional microenvironment element able to modulate growth and other properties of tumour cells. Co-injections of fibroblasts with human epithelial tumoral cells from various tissues have been reported to enhance tumour growth and their metastatic capacity (Picard *et al.*, 1986; Horgan *et al.*, 1987; Camps *et al.*, 1990). These data suggest that several factors including tumour cell-matrix interactions (Liotta, 1984), host cell-tumour cell interactions (Picard *et al.*, 1986; Horgan *et al.*, 1987; Camps *et al.*, 1990; Miller *et al.*, 1988; Price & Zhang, 1990) may affect tumour growth and the metastatic process.

We have investigated the influence of normal human fibroblasts on human breast cancer cells (MCF7 and MDA-MB-231 cells) transplanted into nude mice in the presence or not of matrigel. This report clearly demonstrates that fibroblasts enhance the tumorigenicity of human breast cancer cells *in vivo*. The promoting effects of fibroblasts and matrigel are cumulative. The increased tumorigenicity observed by co-inoculating fibroblasts and tumour cells could be at least partly reproduced by medium conditioned by fibroblasts.

### Materials and methods

### Matrigel

Basement membrane proteins (matrigel) were prepared from dialysed urea extract of EHS (Engelbreth-Holm-Swarm) tumour as previously described (Kleinman *et al.*, 1986; Emonard *et al.*, 1987; Noël *et al.*, 1991). Solid gels were obtained by polymerising 1 ml of this preparation ($10\ mg\ ml^{-1}$) in 35 mm culture dishes, overnight at 37°C in a humid atmosphere.

### Cells

Normal human skin fibroblasts were obtained by outgrowth from explants and used between passages 4 and 12. The human breast carcinoma cell lines, MCF7 cells (Soule *et al.*, 1973) and MDA-MB-231 cells (Cailleau *et al.*, 1974) were kindly provided by Dr Leclercq (Bordet Institute, Brussels, Belgium) and Dr R. Gol (University of Liège, Belgium), respectively. Cells were grown in Dulbecco's Modified Eagles Medium (DMEM) supplemented with 10% foetal calf serum (Gibco), glutamine ($292\ mg\ ml^{-1}$), sodium bicarbonate ($2{,}1\ g\ l^{-1}$), ascorbic acid ($50\ \mu g\ ml^{-1}$) and penicillin-streptomycin ($100\ U\ ml^{-1}$).

### Preparation of conditioned medium (CM)

After three washings to eliminate serum, 8 ml of serum-free medium was added to confluent monolayer of fibroblasts in 10 cm Falcon plastic dishes. The medium was collected after 24 h, centrifuged to eliminate cell debris and used immediately.

### In vivo *studies*

Tumoral cells and/or fibroblasts were detached by trypsinisation, harvested by centrifugation, resuspended in serum-free medium and mixed with 0.25 ml of matrigel ($10\ mg\ ml^{-1}$) in a total volume of 0.5 ml. Cells were injected subcutaneously (SC) into 6 to 8 week-old female athymic (nu/nu) mice (Iffa Credo). The estradiol-dependent MCF7 cells were inoculated into mice previously implanted SC with Silastic capsules (Dow Corning) containing estradiol as previously described (Robinson & Jordan, 1989; Noël *et al.*, 1992*c*). In some assays, MCF7 cells were mixed with matrigel and 0.1 ml of conditioned medium (CM) of fibroblasts. CM (0.1 ml) was reinjected weekly at the site of the primary inoculum and inside the tumours after their appearance.

Injected mice were examined weekly and tumour volume was calculated as previously described (Attia & Weiss, 1966; Noël *et al.*, 1992*c*). The *latency period* was defined as the time between injection and appearance of a 250 $mm^3$ nodule which

Correspondence: A. Noël, Laboratory of Biology, University of Liège, Tour de Pathologie, B23, B-4000 Sart-Tilman, Liège, Belgium.
Received 29 July 1992; and in revised form 11 May 1993.

will maintain a progressive growth. Results are expressed as the mean of the tumour volumes. Tumours presenting a volume lower than 250 $mm^3$ (determining the latency period) were not taken into account because of technical imprecisions of the measurements (Noël *et al.*, 1992*c*).

Each experiment was repeated at least three times (control + experimental variations), at three months intervals, by using different batches of cultured MCF7 cells, fibroblasts and distinct groups of nude mice (Iffa Credo). Each set of animals, in each condition contained at least five to ten individuals. Absolute values of tumour sizes in identical groups could vary as much as 30% between each experiment, probably due to uncontrolled variations (food intake, lighting, seasonal variations, environmental stress, temperature of the unit, . . .). In any case, the absolute trends of variations between the groups in each assay remained consistant and inter-groups variations were of the same extent. The results presented are representative experiment with absolute values ($n = 5-10$). Inter-individual variations of tumour size inside each group ($n = 5-10$) were always lower than 10%.

No death occurred during the course of the experiments. The tumours sizes were always maintained below 1,500 $mm^3$ since such large tumours usually displayed extensive area of central necrosis, ulcerations and subsequent infections and death (data not shown). We therefore decided to finish the assays when appropriate or when the tumours reached 1,250 $mm^3$.

### *Histological examinations and immunohistochemistry*

The tumours were excised, fixed in 10% buffered formalin, embedded in paraffin, sectioned at 4 $\mu m$ intervals and stained with hematoxylin and eosin. The immunohistochemistry was performed as previously described (Noël *et al.*, 1992*c*). For the characterisation of the extracellular matrix, sections were pretreated with pepsin (1 mg $ml^{-1}$ in 0.01 N HCl, 10 min at 37°C). Antiserums directed against vimentin (Calbiochem), fibronectin and types I and III collagen were raised in rabbit (Noël *et al.*, 1992*b*). The anti-Thy antibody, kindly provided by J. Boniver (University of Liège, Belgium) was specific for the murine fibroblasts (Esterre *et al.*, 1992). This species specificity was verified in culture of human and murine fibroblasts.

For transmission electron microscopy, small pieces of tumour tissue (1 $mm^3$) were fixed in 2.5% glutaraldehyde and postfixed in 0.1 M osmium tetraoxide. After dehydration into a graded series of ethanol, samples were embedded in Epon. Ultrathin sections were contrasted with uranyl-acetate and lead-citrate before examination with a JEOL 100 CX II electron microscope (60 kV).

### *Quantification of collagen in tumours*

Tumours were excised, frozen in liquid nitrogen and lyophilised. Their dry weight was then determined. The amount of hydroxyproline was measured by the method of Bergman & Loxley (1963), after hydrolysis in 6 N HCl.

### *Statistical analysis*

Differences between the experimental conditions were evaluated using Student's *t*-test (*P* values lower than 0.02 were considered as significant).

### Results

### *Effect of fibroblasts on human mammary cancer cells tumorigenicity*

Athymic nude mice were inoculated subcutaneously (SC) with estradiol-dependent MCF7 cells in various experimental conditions. In the absence of matrigel, we did not succeed in producing tumours in the 15 nude mice injected with MCF7 cells alone (Noël *et al.*, 1992*a*), even when high number of MCF7 cells ($3 \times 10^6$) were inoculated. However, co-injection of $1 \times 10^6$ fibroblasts with 0.35 or $1.5 \times 10^6$ MCF7 cells resulted in tumour development after approximately 2 months. This promoter effect of human fibroblasts was similar for the two numbers of MCF7 cells inoculated (Figure 1a and b; Table I).

Addition of matrigel induced a more rapid tumour take, even when MCF7 cells were injected, without fibroblasts. Tumours appeared during the first month of observation. The latency period was 22 and 35 days after injection of $1.5 \times 10^6$ and $0.35 \times 10^6$ MCF7 cells, respectively (Table I). Co-injection of fibroblasts with a low number of MCF7 cells ($0.35 \times 10^6$ cells) in the presence of matrigel shortened the latency period (20 *vs* 35 days) (Table I; Figure 1a). The latency period observed for $1.5 \times 10^6$ injected MCF7 cells, was not modified when fibroblasts were added (Table I, Figure 1b). However, independently of the number of MCF7 cells injected simultaneously with fibroblasts, the incidence of tumour always reached 100% and the volume of the tumours was increased (Table I; Figure 1a and b). After 70 days of observation, the volume reached 1,250 $mm^3$ in the presence of fibroblasts and only 750 $mm^3$ in the absence of fibroblasts ($P < 0.01$).

The effect of fibroblasts on the tumorigenicity of an estradiol-independent mammary cancer cell line (MDA-MB-231) was also investigated in the presence of matrigel. Inoculation of $1 \times 10^6$ fibroblasts simultaneously with $0.35 \times 10^6$ MDA cells shortened again the latency period and increased the tumour growth (Figure 2) ($P < 0.02$). The injection of fibroblasts alone used as control in the presence or the absence of matrigel did never induced the development of tumour.

**Figure 1** Effect of co-inoculation of fibroblasts and MCF7 cells upon tumour growth. Tumoral MCF7 cells were injected subcutaneously into nude mice, alone (△), with $1 \times 10^6$ fibroblasts (▲), in the presence of matrigel (○), or in the presence of matrigel and $1 \times 10^6$ fibroblasts (●). The tumour volume was periodically estimated as described in Material and methods. Interindividual variations of tumour sizes inside each group ($n = 5$) were always lower than 10%. The experiment has been repeated three times. **a**, injection of $0.35 \times 10^6$ MCF7 cells with or without $1 \times 10^6$ fibroblasts. **b**, injection of $1.5 \times 10^6$ MCF7 cells with or without $1 \times 10^6$ fibroblasts.

**Table I** Tumorigenicity of MCF7 cells after subcutaneous injection into athymic nude mice in the presence or absence of fibroblasts

| | *Tumorigenicity* $(n/n)^a$ | | *Latency period* $(days)^b$ | |
|---|---|---|---|---|
| | *Without fibroblasts* | *With* $1 \times 10^6$ *fibroblasts* | *Without fibroblasts* | *With* $1 \times 10^6$ *fibroblasts* |
| *Number of MCF7 cells:* in the absence of matrigel | | | | |
| $0.35 \times 10^6$ | 0/5 | 3/5 | – | $70 \pm 3$ |
| $1.5 \times 10^6$ | 0/5 | 3/5 | – | $65 \pm 2$ |
| *Number of MCF7 cells:* in the presence of matrigel | | | | |
| $0.35 \times 10^6$ | 10/15 | 15/15 | $35 \pm 5$ | $20 \pm 2$ |
| $1.5 \times 10^6$ | 8/10 | 10/10 | $22 \pm 2$ | $20 \pm 2$ |

[a]Number of mice bearing tumour larger than 250 $mm^3$/total number of injected mice. [b]Latency period: time between injection and appearance of 250 $mm^3$ nodule.

*Dose-dependence of fibroblasts effects*

When a constant number of MCF7 cells ($0.3 \times 10^6$) was injected with different numbers of fibroblasts (from $0.3 \times 10^6$ to $0.9 \times 10^6$), in the presence of matrigel, the effect on tumour take and on tumour growth (Figure 3) was related to the number of fibroblasts. The effect was maximal at a tumoral cells to fibroblasts ratio of 1 to 2 or 3 ($P < 0.01$).

**Figure 2** Effect of co-inoculation of fibroblasts and MDA-MB-231 cells upon tumour growth, in the presence of matrigel. The MDA-MB231 cells ($0.35 \times 10^6$) were injected into athymic nude mice alone (O) or with $1 \times 10^6$ fibroblasts ( + ), in the presence of matrigel. The tumour volume was measured as described in Material and methods. Interindividual variations of tumour sizes inside each group ($n = 5$) were always lower than 10%. The experiment has been repeated three times.

**Figure 3** Effect of MCF7 cells to fibroblasts ratio or conditioned medium (CM) of fibroblasts upon tumour growth in the presence of matrigel. A constant number of MCF7 cells ($0.3 \times 10^6$) and matrigel were injected without (▲) or with different numbers of fibroblasts (interrupted lines: O = $0.3 \times 10^6$; Δ = $0.6 \times 10^6$; □ = $0.9 \times 10^6$). In one group of mice, MCF7 cells and matrigel were injected in the presence of CM of fibroblasts ( + ). The injection of CM or fibroblasts was repeated weekly, at the site of the inoculum. The volume of tumour was measured as described in Material and methods. Interindividual variations of tumour sizes inside each group ($n = 5$) were always lower than 10%. The experiment has been repeated three times.

*Effect of fibroblasts conditioned medium (CM) on MCF7 cells tumorigenicity*

In an attempt to determine if the promoting effect of fibroblasts results from the production of soluble factors, MCF7 cells suspended in conditioned medium (CM) of fibroblasts were injected with matrigel. This treatment was followed by a weekly injection of CM at the primary site of inoculum. The latency period was not modified as compared to injection of tumoral cells alone (Figure 3). However, after repeated injections of CM, the growth rate and tumour size were increased ($P < 0.01$). After 70 days of observation, the volume reached in these conditions was similar to that obtained after co-inoculation of both cell types at a 1 to 3 MCF7 cells to fibroblasts ratio (Figure 3).

*Light and electron microscopy of tumours*

The histology of tumours developed after injection of MCF7 cells in the presence of matrigel were studied by light and electron microscopy. Tumours appeared to be well circumscribed. Cells were organised into nodules of malignant cells with very few stromal cells (Figure 4a). Numerous mitotic figures were regularly observed. Despite an extensive vascularisation, central necrosis developed. In other areas of the tumour, cells lined up between stromal elements (Figure 4b). The tumours developed after the simultaneous injection of fibroblasts and MCF7 cells in the presence of matrigel were characterised by the regular presence of more abundant stromal structures (Figure 4c).

By electron microscopy, tumour cells presented features of malignant MCF7 cells (high nuclear cytoplasmic ratio, filaments arranged in bundles, numerous mitochondria) (Figure 5a,b). Infiltration of fibroblasts characterised by their abundant rough endoplasmic reticulum was observed in tumours induced by injection of MCF7 cells alone (Figure 5a,b) or in the presence of fibroblasts (Figure 5c,d). In some areas, cells were separated by a granular material resembling matrigel (Kleinman *et al.*, 1986; Noël *et al.*, 1991). Fibrillar striated materials was found at the vicinity of fibroblasts (Figure 5d). Cells were connected to desmosomes-like junctions, interdigitating cytoplasmic projections or the membrane of neighbouring cells were in juxtaposition (Figure 5d). Vascularisation was evidenced in tumours obtained in both conditions (Figure 5e).

*Characterisation of stromal structures*

Since tumours induced by co-inoculation of fibroblasts and tumour cells displayed more stromal structures, the extracellular matrix deposition and stromal cells were characterised by immunohistochemistry. Interstitial collagen types I and III and fibronectin were evidenced in these structures surrounding epithelial tumour cells (Figure 6a). The content of collagen was measured as hydroxyproline present in tumour lysates. The tumours were excised 25 and 40 days after inoculation of MCF7 cells in the presence or absence of fibroblasts. The

**Figure 4** Histology of tumours obtained 1 month after injection of MCF7 cells into athymic nude mice, in the presence of matrigel. MCF7 cells were injected alone **a**,**b**, in the presence of fibroblasts **c**, or with CM of fibroblasts **d**, as described in Materials and methods (bar = 100 μm).

collagen content of tumours developed after co-inoculation of both cell types was double that of tumours induced by injection of MCF7 cells alone ($92 \pm 20\,\mu g$ collagen $mg^{-1}$ dry weight *vs* $50 \pm 10\,\mu g$ collagen $mg^{-1}$ dry weight; $P < 0.005$). Fibroblasts stained positively for vimentin. Surprisingly, the fibroblasts as shown by using anti-Thy 1 antibodies revealed to be exclusively of murine origin (Figure 6b). This antibody is specific for murine fibroblasts and not for human fibroblasts, as verified on our fibroblasts in culture (data not shown). All stromal cells stained positively with this antibody.

Similar histological features were observed after repeated injections of CM of fibroblasts, at the site of inoculation of MCF7 cells with matrigel (Figure 4d).

### Discussion

In this study, we demonstrate that the addition of fibroblasts to MCF7 cells which are not tumorigenic by themselves allows the take and growth of tumours. These data confirm the helper effect of normal fibroblasts as observed for different types of tumour cells of animal and human origin derived from breast (Horgan *et al.*, 1987; Camps *et al.*, 1990), rhabdomyosarcoma (Picard *et al.*, 1986), melanoma (Tanaka *et al.*, 1988), prostate and bladder (Camps *et al.*, 1990). However, tumours induced by co-injection of MCF7 cells and fibroblasts appeared only after 2 months, in a rather small proportion of injected animals and grew slowly (Table I and Figure 1). More recently, matrigel has been shown to accelerate tumour growth in athymic nude mice (Fridman *et al.*, 1990; 1991; Pretlow *et al.*, 1991). We observed similar results since tumours developed 2 to 4 weeks after injection of MCF7 cells in the presence of matrigel (Noël *et al.*, 1992*c*). The exact mechanisms operating in the enhancement of tumorigenicity by matrigel are not clear and have been previously discussed (Fridman *et al.*, 1990; 1991; Noël *et al.*, 1992*c*). The effect of matrigel on the human cells tumorigenicity was partially abolished by the addition of a

**Figure 5** Electron micrographs of tumours obtained after injections of MCF7 cells in the presence of matrigel into athymic nude mice. **a,b**, Tumour obtained after injection of MCF7 cells (M) alone. Rare presence of fibroblasts (F) was observed (bar = 1 μm). **c–d**, Tumour obtained after injection of MCF7 cells (M) and fibroblasts. Fibroblasts (F) were surrounded by striated fibrillar material (c), (bar = 1 μm). Cells were connected by desmosome (arrow) or interdigitating cytoplasmic projections (d) (bar = 0.5 μm). **e**, Capillary surrounded by extracellular matrix and tumoral MCF7 cells. (bar = 100 μm).

synthetic peptide from the B1 chain of laminin (Fridman *et al*., 1990) suggesting that it could be at least partly ascribed to specific cell-matrix interactions promoted by laminin (Noël *et al*., 1988, 1993).

When fibroblasts were inoculated simultaneously with a low number of MCF7 cells in the presence of matrigel, the latency period was reduced and the tumour growth was increased. These effects are correlated with the ratio of tumoral cells to fibroblasts. In these conditions, all mice developed tumours. Similar results were obtained with an other breast cancer cell line (MDA-MB-231) and are not restricted to the MCF7 cell line. The factors supplied by fibroblasts and matrigel are cumulative as shown by the results obtained with a low number of tumoral cells. Furthermore, injections of higher amounts of matrigel or repeated injections of matrigel were unable to mimic the effect of fibroblasts (data not shown). These results suggest that fibroblasts and matrigel enhance tumour growth by distinct mechanisms. Identical tumour sizes were obtained when $0.3 \times 10^6$ or $3 \times 10^6$ MCF7 cells were inoculated indicating that the tumour growth was not proportional to the number of MCF7 cells injected. The identical latency periods observed when $1.5 \times 10^6$ MCF7 cells were injected in the presence of matrigel, with or without fibroblasts suggest that the latency period is minimal or that local host factors limit the rate of tumour take.

Histological observations demonstrated the presence of fibroblasts and stromal-like structures inside of the tumour islets. This stroma contained fibronectin, collagen types I and III as evidenced by immunohistochemistry. The content of total collagen was twice as high in tumours induced by co-inoculation of tumour cells and fibroblasts. This could be ascribed at least partly to a modulation of collagen production by fibroblasts in response to MCF7 cells. We have indeed demonstrated the capacity of these mammary tumour cells to secrete soluble factors which enhance collagen synthesis by fibroblasts *in vitro* (Noël *et al*., 1992*a*,*b*).

The murine origin of fibroblasts surrounding epithelial

**Figure 6** Immunoperoxidase stainings of tumours obtained after injections of MCF7 cells and fibroblasts, in the presence of matrigel into athymic nude mice. **a**, Immunostaining with a polyclonal antibody localises the collagen type I in the stroma surrounding tumour cells. **b**, Immunostaining of fibroblasts with anti-Thy 1 antibody raised against human fibroblasts antigen. (bar = 1 μm)

tumour nests was determined using an antibody staining specifically murine fibroblasts. Furthermore, the identical histological features of tumours induced by addition of fibroblasts or by injections of medium conditioned by fibroblasts indicate recruitment of murine fibroblasts rather than a persistance of the injected human fibroblasts. In this regard, fibroblasts have been shown to secrete several factors affecting cell motility (for review, Stocker & Gherardi, 1991).

Various mechanisms may be involved in the fibroblasts-mediated increase of tumorigenicity. Camps *et al.* (1990) reported that lethally irradiated fibroblasts retain at least part of their potential to accelerate tumour growth, suggesting the involvement of the bio matrix surrounding the cells. This effect could be ascribed to specific properties of its components or to its capacity to bind growth factors (Nathan & Sporn, 1991). For example, several forms of fibroblasts growth factors (FGF) are deposited in the extracellular matrix and function when bound to proteoglycans of the extracellular matrix (Nathan & Sporn, 1991; Bashkin *et al.*, 1989). According to previous observations (Picard *et al.*, 1986) and to our results, fibroblasts conditioned medium (CM) mixed with tumour cells also stimulates tumour growth (Figure 3). The failure of CM to shorten the latency period could be ascribed to subcutaneous dilution of soluble factors before tumour take (Gärtner *et al.*, 1992). Nevertheless, these data suggest the role of soluble growth factors secreted by fibroblasts. The production of IGF-I and IGF-II (Clemmons, 1984; Yee *et al.*, 1988) by skin fibroblasts might be responsible for this paracrine effect (Van Roozendaal *et al.*, 1992). Furthermore, the matrigel, known to bind several cytokines *via* the heparan sulfate proteoglycan (Noël *et al.*, 1992*a*; Taub *et al.*, 1990; Vukicevic *et al.*, 1992) could act as a 'reservoir' accumulating these intercellular messengers.

The maximal stimulation is obtained when the tumour cells are in contact with fibroblasts. It is likely that maximal stimulation of tumorigenicity requires the continuous production of factors by fibroblasts and cell-cell contacts. The importance of contacts between tumour cells and fibroblasts have been suggested by several studies (Gärtner *et al.*, 1992; Tanaka *et al.*, 1988; Coucke *et al.*, 1992). The lung colonising potential of a low metastatic clone of melanoma cells was indeed increased when cells were cocultured *in vitro* with fibroblasts. The CM of cocultured melanoma cells and fibroblasts presented similar potential whereas medium from monoculture of fibroblasts showed only a low activity (Tanaka *et al.*, 1988). We have previously demonstrated that matrigel promotes not only cell-matrix interactions but also cell-cell interactions in MCF7 cells culture (Noël *et al.*, 1988). The same gel also operated in tumour cell-fibroblasts interactions since in coculture *in vitro*, MCF7 cells organised into clusters attached on top of fibroblasts aggregates (Noël *et al.*, 1993). These interactions are modulated by both the soluble and insoluble forms of laminin and fibronectin. In addition, when cultured on matrigel, fibroblasts have been shown to deposit extracellular material, resulting in a progressively more fibrillar pattern of the matrix gel (Emonard *et al.*, 1987). Fibroblasts are also known to actively organise the network of interstitial type I and III fibrils (Bell *et al.*, 1979). Such rearrangements of the tissue architecture might also modulate tumour growth.

In conclusion, our results emphasise the importance of tumour-host interactions and mainly basement membrane proteins and fibroblasts or their synthetic products on cells tumorigenicity. Our work also suggests that human tumours may be reconstituted and grown in athymic nude mice using stromal and basement membrane components as inducers. This model may be helpful in the study of factors mediating cellular interactions during neoplastic progression and for testing anticancer agents.

We gratefully acknowledge the excellent technical assistance of Mrs P. Gavitelli, Mrs M.J. Nix and Mr G. Roland. We thank Mrs E. Welliquet for her skillful typographic help during the preparation of the manuscript and Mr L. Duchâteau for performing the photographic work. This study was supported by grants of the 'Fond Cancérologique de la CGER', the 'Loterie nationale' (no 9.4541.92), the 'Centre Anticancéreux près l'Université de Liège' (Belgium), the Faculty of Medecine (University of Liege, Belgium), the 'Association Sportive Contre le Cancer' (Belgium), the 'Fond National de la Recherche Scientifique' and the 'Communauté Française de Belgique' ('Action de Recherche Concertée'n no 90/94-139).

### References

ATTIA, M.A. & WEISS, D.W. (1966). Immunology of spontaneous mammary carcinomas in mice. V: acquired tumor resistance and enhancement in strain a mice infected with mammary tumor virus. *Cancer Res.*, **26**, 1787–1800.

BASHKIN, P., DOCTROW, S., KLAGBURN, M., SVAHN, C.M., FOLKMAN, J. & VLODAVSKY, I. (1989). Basic fibroblasts growth factor binds to subendothelial extracellular matrix and is released by heparinase and heparin-like molecules. *Biochemistry*, **28**, 1737–1743.

BELL, E., IVARSSONS, B. & MERRIL, C. (1979). Production of a tissue-like structure by contraction of collagen lattices by human fibroblasts of different proliferative potential *in vitro*. *Proc. Natl Acad. Sci. USA*, **76**, 1274–1278.

BERGMAN, I. & LOXLEY, R. (1963). Two improved and simplified methods for the spectrophotometric determination of hydroxyproline. *Anal. Chem.*, **35**, 1961–1964

CAILLEAU, R., YOUNG, R., OLIVE, M. & REEVES, W.J. (1974). Breast tumor cell lines from pleural effusions. *J. Natl Cancer Inst.*, **53**, 661–674.

CAMPS, J.L., CHANG, S.M., HSU, T.C., FREEMAN, M.R., HONG, S.J., ZHAU, H.E., VON ESCHENBACH, A.C. & CHUNG, L.W.K. (1990). Fibroblast-mediated acceleration of human epithelial tumor growth *in vivo*. *Proc. Natl Acad. Sci. USA*, **87**, 75–79.

CLEMMONS, D.R. (1984). Multiple hormones stimulate the production of somatomedin by cultured human fibroblasts. *J. Clin. Endocr. Metab.*, **58**, 850.

COUCKE, P., DE LEVAL, L., LEYH, PH., BONJEAN, K., SIWEK, B., NOEL, A., DE PAUW-GILLET, M.CL., PAULUS, J.M., BASSLEER, R. & FOIDART, J.M. (1992). Influence of laminin or fibroblasts upon colony formation in the mouse by B16 melanoma cell spheroids: a morphometric analysis. *In vivo*, **6**, 119–124.

EMONARD, H., CALLE, A., GRIMAUD, J.A., PEYROL, S., CASTRONOVO, V., NOËL, A., LAPIÈRE, CH.M., KLEINMAN, H. & FOIDART, J.M. (1987). Interactions between fibroblasts and a reconstituted basement membrane matrix. *J. Invest. Dermatol.*, **89**, 156–163.

ESTERRE, P., MELIN, M., SERRAR, M. & GRIMAUD, J.A. (1992). New specific markers of human and mouse fibroblasts. *Cell. & Mol. Biol.*, **38**, 297–301.

FRIDMAN, R., GIACCONE, G., KANEMOTO, T., MARTIN, G.R., GAZDAR, A.F. & MULSHINE, J.L. (1990). Reconstituted basement membrane (matrigel) and laminin can enhance the tumorigenicity and the drug resistance of Small Cell Lung Cancer cell lines. *Proc. Natl Acad. Sci. USA*, **87**, 6698–6702.

FRIDMAN, R., KIBBEY, M.C., ROYCE, L.S., ZHAIN, M., SWEENEY, T.M., JICHA, D.L., YANNELLI, J.R., MARTIN, G.R. & KLEINMAN, H.K. (1991). Enhanced tumor growth of both primary and established human and murine tumor cells in athymic mice after coinjection with matrigel. *J. Natl Cancer Inst.*, **83**, 769–774.

GARTNER, M., WILSON, L. & DOWDLE, E.B. (1992). Fibroblast-dependent tumorigenicity of melanoma xenograft in athymic mice. *Int. J. Cancer*, **51**, 788–791.

HORGAN, K., JONES, D.L. & MANSEL, L. (1987). Mitogenicity of human fibroblasts *in vivo* for human breast cancer cells. *Br. J. Surg.*, **74**, 227–229.

KLEINMAN, H.K., MCGARVEY, M.L., HASSELL, J.R., STAR, V.L., CANNON, F.B., LAURIE, G.W. & MARTIN, G.R. (1986). Basement membrane complexes with biological activity. *Biochemistry*, **25**, 312–318.

LIOTTA, L.A. (1984). Invasion and metastasis: role of basement membrane. *Am. J. Pathol.*, **117**, 339–348.

MILLER, F.R. & MCINERNEY, D. (1988). Epithelial component of host-tumor interactions in the orthotopic site preference of a mouse mammary tumor. *Cancer Res.*, **48**, 3698–3701.

NATHAN, C. & SPORN, M. (1991). Cytokines in context. *J. Cell. Biol.*, **113**, 981–986.

NOËL, A., CALLÉ, A., EMONARD, H., NUSGENS, B., FOIDART, J.M. & LAPIÈRE, CH. M. (1988). Antagonistic effects of laminin and fibronectin in cell to cell and cell to matrix interactions in MCF7 cultures. *In vitro*, **24**, 373–379.

NOËL, A., CALLÉ, A., EMONARD, H., NUSGENS, B., FOIDART, J.M. & LAPIÈRE, CH.M. (1991). Lack of invasion of reconstituted basement membrane matrix by tumor cells. *Cancer Res.*, **51**, 405–414.

NOËL, A., MUNAUT, C., NUSGENS, B., FOIDART, J.M. & LAPIÈRE, CH.M. (1992*a*). The stimulation of fibroblasts collagen synthesis by neoplastic cells is modulated by the extracellular matrix. *Matrix*, **12**, 213–220.

NOËL, A., MUNAUT, C., BOULVAIN, A., CALBERG-BACQ, C.M., LAMBERT, CH.A., NUSGENS, B., LAPIERE, CH.M. & FOIDART, J.M. (1992*b*). Modulation of collagen and fibronectin synthesis in fibroblasts by normal and malignant cells. *J. Cell. Biochem.*, **48**, 150–161.

NOËL, A., SIMON, N., KLEINMAN, H.K., RAUS, J. & FOIDART, J.M. (1992*c*). Basement membrane components (matrigel) promote human breast adenocarcinoma MCF7 cells tumorigenicity and provide an *in vivo* model to assess cell responsiveness to estrogen. *Biochem. Pharmacol.*, **43**, 1263–1267.

NOËL, A., NUSGENS, B., LAPIÈRE, CH.M. & FOIDART, J.M. (1993). Interaction between tumoral MCF7 cells and fibroblasts on matrigel and purified laminin. *Matrix*, (in press).

PICARD, O., ROLLAND, Y. & POUPON, M.F. (1986). Fibroblast-dependent tumorigenicity of cells in nude mice: implication for implantation of metastases. *Cancer Res.*, **46**, 3290–3295.

PRETLOW, T.G., DELMORO, C.M., DILLEY, G.G., SPADAFORA, C.G. & PRETLOW, T.P. (1991). Transplantation of human prostatic carcinoma into nude mice in matrigel. *Cancer Res.*, **51**, 3814–3817.

PRICE, J.E. & ZHANG, R.D. (1990). Studies of human breast cancer metastasis using nude mice. *Cancer Metast. Rev.*, **8**, 285–297.

ROBINSON, S.P. & JORDAN, V.C. (1989). Antiestrogenic action of toremifene on hormone-dependent, -independent, and heterogeneous breast tumor growth in the athymic mouse. *Cancer Res.*, **49**, 1758–1762.

SOULE, H.D., VAZQUEZ, J., LONG, A., ALBERT, S. & BRENNAN, M.T. (1973). Human cell line from pleural effusion derived from breast carcinoma. *J. Natl Cancer Inst.*, **51**, 1409–1413.

STOKER, M. & GHARARDI, E. (1991). Regulation of cell movement: the mitogenic cytokines. *B.B.A.*, **1072**, 81–102.

TANAKA, H., MORI, Y., ISHII, H. & AKEDO, H. (1988). Enhancement of metastatic capacity of fibroblast-tumor cell interaction in mice. *Cancer Res.*, **48**, 1456–1459.

TAUB, M., WANG, Y., SZCZESNY, T.M. & KLEINMAN, H. (1990). Epidermal growth factor or transforming growth factor alpha is required for kidney tubulogenesis in matrigel cultures in serum-free medium. *Proc. Natl Acad. Sci. USA*, **87**, 4002–4006.

VAN ROOZENDAAL, C.E.P., VAN OOIJEN, B., KLIJN, J.G.M., CLAASSEN, C., EGGERMONT, A.M.M., HENZEN-LOGMANS, S.C. & FOEKENS, J.A. (1992). Stromal influences on breast cancer cell growth. *Br. J. Cancer*, **65**, 77–81.

VUKICEVIC, S., KLEINMAN, H., LUYTEN, F.P., ROBERTS, A.B., ROCHE, N.S. & REDDI, A.H. (1992). Identification of multiple active growth factors in basement membrane matrigel suggests caution in interpretation of cellular activity related to extracellular matrix components. *Exper. Cell Res.*, **202**, 1–8.

YEE, D., CULLEN, K.J., PAIK, S. & OTHERS (1988). Insulin-like growth factor II mRNA expression in human breast cancer. *Cancer Res.*, **48**, 6691–6704.